# Effects of Short- and Long-Term Variations in RLS Severity on Perceived Health Status – the COR-Study

**DOI:** 10.1371/journal.pone.0094821

**Published:** 2014-04-17

**Authors:** Andrea Fuhs, Dunya Bentama, Rafael Antkowiak, Johannes Mathis, Claudia Trenkwalder, Klaus Berger

**Affiliations:** 1 Institute of Epidemiology and Social Medicine, University of Muenster, Muenster, Germany; 2 Department of Neurology, Inselspital, Bern University Hospital, Bern, Switzerland; 3 Department of Neurology, Paracelsus-Elena-Klinik Kassel, Kassel, Germany; University of Ulm, Germany

## Abstract

In a cohort study among 2751 members (71.5% females) of the German and Swiss RLS patient organizations changes in restless legs syndrome (RLS) severity over time was assessed and the impact on quality of life, sleep quality and depressive symptoms was analysed. A standard set of scales (RLS severity scale IRLS, SF-36, Pittsburgh Sleep Quality Index and the Centre for Epidemiologic Studies Depression Scale) in mailed questionnaires was repeatedly used to assess RLS severity and health status over time and a 7-day diary once to assess short-term variations. A clinically relevant change of the RLS severity was defined by a change of at least 5 points on the IRLS scale. During 36 months follow-up minimal improvement of RLS severity between assessments was observed. Men consistently reported higher severity scores. RLS severity increased with age reaching a plateau in the age group 45–54 years. During 3 years 60.2% of the participants had no relevant (±5 points) change in RLS severity. RLS worsening was significantly related to an increase in depressive symptoms and a decrease in sleep quality and quality of life. The short-term variation showed distinctive circadian patterns with rhythm magnitudes strongly related to RLS severity. The majority of participants had a stable course of severe RLS over three years. An increase in RLS severity was accompanied by a small to moderate negative, a decrease by a small positive influence on quality of life, depressive symptoms and sleep quality.

## Introduction

Restless legs syndrome is a common sleep disorder with prevalences between 5 and 10% in population based studies in Europe and North America [1]–[4].

The prevalence of RLS increases with age, while the course of the disease, i.e. the pattern of symptom severity over time, is unclear. Assessing the latter has been predominantly done in clinical trials testing specific single medication over follow-up periods between one week and 35 months [5]. Thus, it is unknown if RLS is a chronic condition progressing slowly after onset over the life span or expresses other patterns over long time periods in individual patients depending on life events or associated diseases.

Knowledge about the disease course is important because many studies have shown that RLS influences different aspects of daily living when assessed in cross-sectional studies [6]. Beside reductions in sleep quality, activities of daily living and quality of life it includes a 2- to 4-fold risk for depressive disorders in RLS patients compared to healthy individuals [7], [8]. However, studies assessing symptom severity in affected individuals repeatedly over longer time periods are lacking. Thus, the basis for patient information on the long-term prognosis is limited to the experience of the respective physician with long term treatment. Better knowledge of short-term fluctuations and long-term severity patterns however might enable an individual to better cope with the consequences of the disease.

Aim of this analysis was to evaluate individual variations of RLS severity over a short, i.e. 7-day, and a long, i.e. 36-month time-period and to analyse the impact of these variations on self perceived quality of life, sleep quality and depressive symptoms in a cohort of German and Swiss RLS patients.

## Methods

### Subjects

The Course of RLS-Study (COR-S) was started in fall 2007 and is conducted by mailed questionnaires. All 4385 members of the RLS support-group in Germany (RLS e.V. Deutsche Restless Legs Vereinigung) and the 633 members of the Swiss RLS Patient Association (Schweizerische Restless Legs Selbsthilfegruppe), were once contacted by mail, and invited to participate in the study. 2562 members of the German Restless Legs group (response proportion 58.4%), and 254 members of the Swiss support group (response proportion 40.1%) accepted the invitation, yielding a COR-S population of 2816 participants (overall response 56.1%). Written informed consent was obtained from all participants. A small proportion (6.7%) of the German participants had undergone a standardized, detailed diagnostic work up in one of 5 specialized German RLS centers. Participants from these ARELESS centers underwent a physical examination including a complete neurological status assessment by an RLS expert. Laboratory markers including ferritine were analysed. Optionally, a polysomnography was done. Inclusion of these participants enabled the definition of a RLS group that had received a common diagnostic ‘gold standard’ procedure in the establishment of the RLS diagnosis. This analysis includes data from the baseline questionnaire (Q1) and the six- (Q2), 12- (Q3), 24- (Q4) and 36-months follow-up questionnaires. During this time period a total of 78 participants had died.

### Instruments

#### Ethics statement

The study protocol was approved by the local ethics committee of the Medical Faculty at the University of Muenster.

In all five questionnaires a common set of instruments and scales was used to assess RLS severity and different aspects of self-perceived health status. All scales applied in the respective German version had been validated in prior studies and are frequently used in RLS studies.

To assess the severity of RLS the International RLS Study Group (IRLSSG) rating scale for severity of RLS (IRLS) was used [9]. An IRLS score between 1 and 10 points is considered as mild, between 11 and 20 moderate, between 21 and 30 severe and between 31 and 40 points as very severe RLS.

In the 6-month questionnaire (Q2) a 7-day diary was included to assess short-term variations in the two key RLS symptoms “dysaesthesia” and “urge to move”. Splitting every day into four time periods the RLS symptoms were rated by the participants in each time period as either being mild or moderate or severe.

To assess the self perceived health status the German version of the Short Form-36 Health Survey (SF-36) was used [10].

Sleep related problems were assessed with the Pittsburgh Sleep Quality Index (PSQI) [11]. Varying between 0 and 21 points a PSQI summary score ≥5 is considered to indicate sleep disturbances.

Depressive symptoms were rated using the Center for Epidemiological Studies Depression Scale (CES-D) [12]. A cut-off of ≥16 points is frequently used to identify clinical relevant depression, i.e. an acute episode of major depression.

In all COR-S questionnaires information on sociodemographic factors, health related risk factors, comorbidities and health services utilization was collected.

### Statistical analysis

To analyse changes in health status scores data of four time points, baseline questionnaire (Q1), 12- (Q3), 24- (Q4) and 36-months were applied to assess one-way repeated measures ANOVA. In addition we used the difference in the RLS severity score between baseline and 36-months to define long-term change. Following arguments by Trenkwalder [13] we defined as a “clinically relevant change” a decrease or increase of more than 5 points on the IRLS. This cut-off is a conservative definition, since some RLS drugs have been licensed with a minimum change of 3 points difference in the IRLS compared to placebo after 12 weeks of therapy [13]. Subsequently one way analysis of variance (ANOVA) was applied to test for differences in the means of the respective health status scores at each time point, according to three levels of change (improvement, unchanged, worsening). Multivariable linear regression was performed to analyse the impact of change in RLS severity on changes in sleep quality, quality of life and depression scores. These models were adjusted for age at baseline, gender, duration of RLS disease and number of RLS medications at baseline. A p-value <0.05 was considered statistically significant. All analyses were done with STATA 9.0 (StatCorp LP, College Station, TX, USA).

## Results

In total 2751 patients were recruited into the COR-Study. Table 1 summarizes characteristics of the study population at baseline and life time prevalences of self reported physician-diagnosed comorbidities.

**Table 1:** Characteristics of the COR-Study participants at baseline.

Baseline questionnaire(Q1)	all	women	men
Sex, n (%)	2751 (100)	1967 (71.5)	783 (28.5)
Mean age (range), years	65.9 (27–96)	65.5 (29–96)	66.9 (27–94)
RLS history			
Duration of RLS symptoms, years mean	22.1	22.7	20.7
Time since diagnosis, years mean	8.3	8.3	8.5
RLS diagnosed by a neurologist,%	60.1	59.7	61.1
RLS treated by a neurologist,%	71.0	71.4	70.0
Medication			
RLS medication use,%	93.8	93.9	93.7
Other medication use,%	85.6	87.7	80.1
No medication at all,%	1.4	1.3	1.8
Smoking habits			
Current smokers,%	9.6	10.0	8.6
Former smokers,%	30.3	24.1	45.9
Alcohol consumption			
Non drinker,%	35.5	39.5	32.2
Mean intake among drinkers, g/day	16.3	13.0	22.3
Body mass index ≥30 kg/m^a^,%^1^	18.8	19.9	16.3
Comorbidities			
History of hypertension^b^,%	46.8	46.4	47.7
History of depression^b^,%	29.6	31.8	24.0
History of cancer^b^,%	12.5	12.1	13.5
History of diabetes^b^,%	11.0	10.1	13.0
History of myocardial infarction^b^,%	5.1	3.3	9.4
History of stroke^b^,%	3.4	3.2	3.0
Additional of comorbidities to RLS^b^	2.6	2.6	2.4
1 additional comorbidity^b^,%	18.3	17.6	19.8
2 additional comorbidities^b^,%	24.3	24.9	22.7
≥5 additional comorbidities^b^,%	14.2	14.8	12.8

a based on self-reported weight and height.

b self-reported physician diagnosis.

From a list of 17 comorbidities, partly important as being related to RLS, 11.2% of the participants reported no additional comorbidity at baseline. Complete information for the IRLS in all five questionnaires was provided by 84.2% of the participants. Mean IRLS scores differed only slightly between the five assessments (Table 2), with men consistently reporting higher scores.

**Table 2:** Mean values in RLS severity (IRLS), quality of life (SF-36), depression (CES-D) and sleep (PSQI) scales at baseline 12-months, 24-months and 36-months follow-up.

Scale	Baseline (Q1)	12-months F-up (Q3)	24-months F-up (Q4)	36-months F-up (Q5)	*p-value* a
RLS severity scale (IRLS), mean	25.2	25.2	23.2	23.0	<0.01
Short-Form 36 Health Survey (SF-36), means					
Physical Functioning (PF)	64.1	65.0	62.9	60,3	<0.01
Role Physical (RP)	50.1	50.5	47.4	46.9	<0.01
Role Emotional (RE)	67.7	68.4	67.1	66.2	<0.01
Social Functioning (SF)	68.6	69.0	68.6	67.3	<0.01
Mental Health (MH)	63.2	63.2	62.4	62.2	<0.01
Bodily Pain (BP)	50.4	50.5	50.3	50.6	0.22
Vitality (VT)	46.6	47.1	47.2	45.8	<0.01
General Health (GH)	49.9	49.7	50.9	50.1	0.05
Physical Component Summary	39.2	39.3	38.9	38.4	<0.01
Mental Component Summary	46.1	46.2	46.2	45.8	0.24
Center for Epidemiologic Studies Depression Scale (CES-D)	17.9	17.7	17.8	18.1	0.07
Depressive affect	4.1	4.2	4.1	4.3	0.04
Somatic symptoms	6.5	6.5	6.6	6.7	<0.01
Positive affect	6.6	6.5	6.5	6.6	0.23
Interpersonal relations	0.4	0.4	0.4	0.4	0.03
Pittsburgh Sleep Quality Index (PSQI)	10.3	10.4	10.6	10.7	<0.01

a p-value for difference between assessments according to one-way repeated measures ANOVA.

**Figure 1:**
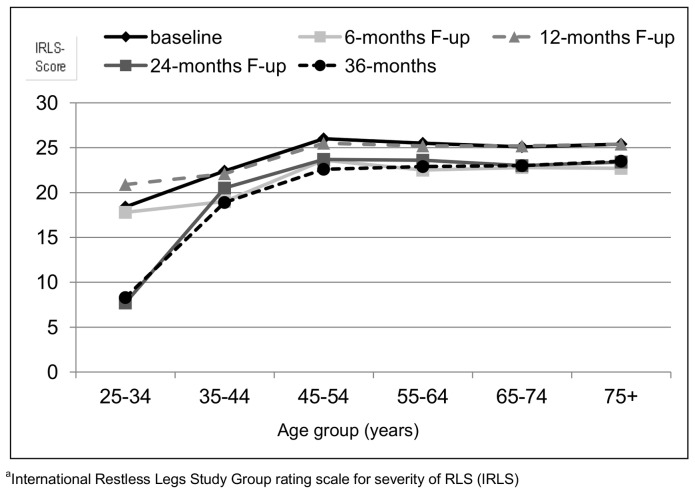
Mean IRLS^a^ score according to six age groups at baseline, 6-, 12- 24- and 36-months follow-up.

Across all five assessments RLS severity increased with age up to the age group of 45–54 years (figure 1) but not further.

**Figure 2:**
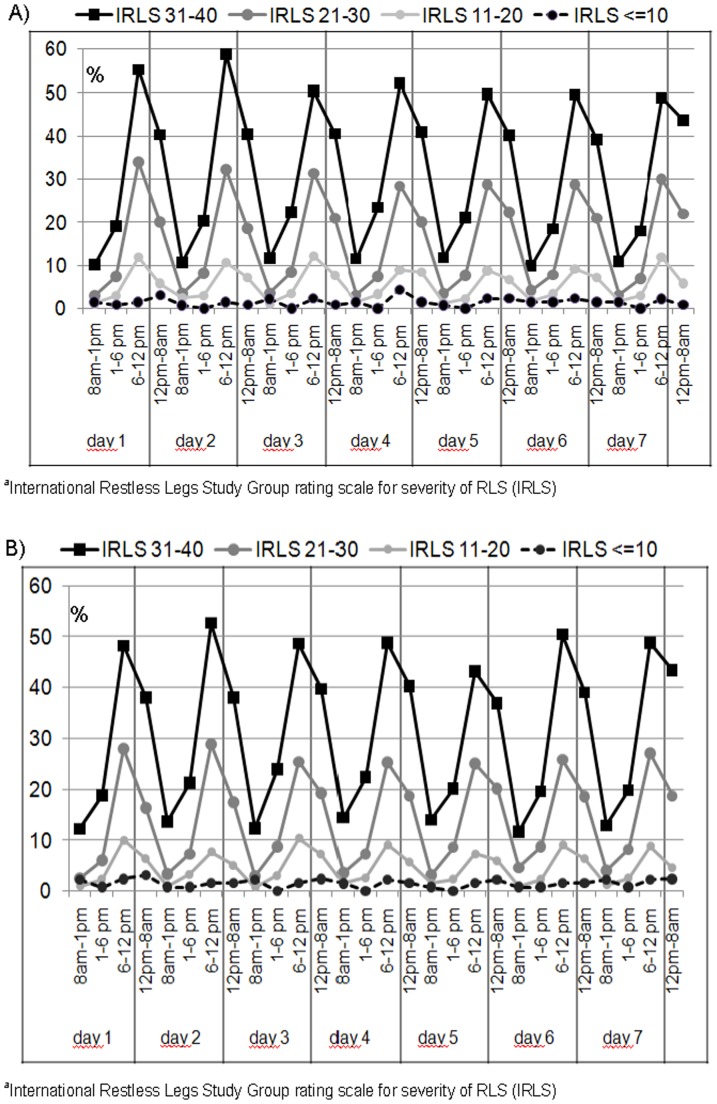
A) Short term variation of the RLS minimal criterion “urge to move” in the 7-day diary, according to IRLS^a^ score and time of the day. B) Short term variation of the RLS minimal criterion “dysaesthesia” in the 7-day diary according IRLS^a^ score and time of the day.

Figures 2a and b show self-reported daily variations in the severity of the two key symptoms of RLS, “urge to move” and “dysaesthesia”, implemented in the 7-day diary of Q2 (6-months follow-up). The figures summarize participant perceptions of these two symptoms as being “severe” during four time periods every day. The four lines in each figure represent participants in the RLS categories of mild, moderate, severe and very severe, derived from the IRLS summary score. Subjects with severe and very severe RLS particularly reported a distinct circadian pattern for both symptoms with expected peaks in the time periods 6.00 p.m.to midnight and midnight to 8.00 a.m.

Table 2 shows changes in mean scores of RLS severity-, self-perceived health status- (SF-36), depressive symptom scores (CES-D) and sleep related problems (PSQI) in the four assessments. The mean IRLS score decreased by 2.2 points over the 3 years course. The SF-36 subscore changes were small, resulting in small decreases in both the physical- and the mental component summary score.

At baseline 53.2% of the participants of the COR-Study had a score of ≥16 on the CES-D indicating a high likelihood for an ongoing episode of clinical depression. The mean CES-D score over time remained unchanged and was constantly higher in women than in men. 30.9% of the study participants had persistent high CES-D scores indicating a high likelihood of a clinical diagnosis of major depression. Depressive symptoms were strongly related to RLS severity, increasing from a CES-D score of 11.3 among those with mild symptoms (≤10 points IRLS) to 24.5 in those with high severity (>30 points IRLS), after controlling for age and gender (not shown). Average PSQI-scores increased slightly over the course of 3 years, indicating a small worsening in sleep quality.

Applying our definition of ‘clinically relevant change’ (IRLS±5 points), 27.3% of the participants of the COR-Study “improved”, 12.5% “worsened” and 60.2% of the subjects showed “no change” over the course of three years. Table 3 describes characteristics in health status scores in these three groups according to the time of assessment.

**Table 3:** Clinical characteristics and differences in health status according to changes in RLS severity (IRLS^c^) over 36 months.

	36 months change^b^ in RLS severity			
	Worsening 12.5%	Unchanged 60.2%	Improvement 27.3%	*p-value*
Age at baseline, years	65.8	65.5	64.6	0.03*
Women, %	78.3	71.9	71.2	0.06^a^
Men; %	21.7	28.1	28.8	
Mean IRLS^3^ score at baseline	16.0	25.3	28.4	<0.01*
Mean IRLS^3^ score at 6-months follow-up	19.7	23.5	21.9	<0.01*
Mean IRLS^3^ score at 12-months follow-up	23.5	25.7	24.4	<0.01*
Mean IRLS^3^ score at 24-months follow-up	22.2	24.5	20.8	<0.01*
Mean IRLS^3^ score at 36-months follow-up	26.5	25.1	16.6	<0.01*
Center for Epidemiologic Studies Depression Scale (CES-D)				
Mean score baseline	14.6	17.9	18.4	<0.01*
Mean score 36-months follow- up	18.8	18.7	16.3	<0.01*
Pittsburgh Sleep Quality Index (PSQI)				
Mean score baseline	8.7	10.6	10.5	<0.01*
Mean score 36-months follow-up	10.3	11.1	9.8	<0.01*
Physical Component Summary (PCS of SF-36)				
Mean score baseline	41.3	39.5	39.8	0.07*
Mean score 36-months follow-up	38.4	37.7	40.0	<0.01*
Mental Component Summary (MCS of SF-36)				
Mean score baseline	49.1	45.9	45.8	0.01*
Mean score 36-months follow-up	45.8	45.2	47.1	0.01*

*p for differences between the 3 groups derived from one-way analysis of variance (ANOVA) unless otherwise noted.

a Chi-square test for the difference between gender.

b Change in the International Restless Legs Study Group (IRLSSG) Rating Scale for severity of RLS between baseline and 36 months follow-up: worsening = increase in score by >5 points, unchanged = change in score not more than ±5 points, improving =  decrease in score >5 points.

c International Restless Legs Study Group Rating Scale for severity of RLS (IRLS).

Small variations indicate a slight worsening of the CES-D- and PSQI scores as well as the physical and mental component summary scores of the SF-36 among those with “unchanged” RLS severity. In contrast, the “worsened” group reported considerable worse scores in all categories. The change of mean RLS severity over time followed a rather consistent trend across the five points of assessment. Those with a worsening of symptoms had a rather low RLS severity level at baseline while the group with improved symptom severity started at a relatively high level (28.4 IRLS points). Compared to the changes in the IRLS, the changes in the depression, quality of life and sleep scales were rather small.

In multivariable linear regression analyses we analysed the effect of a change in IRLS between baseline and 36-months follow-up on the changes in CES-D-, PSQI and the SF-36 summary scores in separate models, adjusted for gender, age, number of comorbidities, RLS duration and the specific scale's baseline score. An increase of the IRLS score by one point yielded a significant increase of the CES-D score by 0.21 points (95% CI, 0.17–0.25). The first also yielded a significant increase of the PSQI by 0.13 points (95% CI, 0.11–0.15) and significant decreases in the physical (−0.12; 95% CI, −0.17–−0.09) and mental component summary scores (−0.16; 95% CI, −0.20–−0.11) of the SF-36 (not shown).

## Discussion

The Course of RLS (COR-S) Study analysed short (7-day) and long-term (36-months) variations in RLS severity over time and their influence on quality of life, depression and sleep quality. Among participants an average improvement of less than 1 point in RLS severity per 12 months was observed during 3 years of follow-up. We consider this annual change as clinically not relevant. RLS severity increased with age reaching a plateau in the age group 45–54 years in all assessments. This finding indicates a stable average severity level at least under medical treatment from midlife on. Thus, our results do not support the hypothesis that “RLS worsens with age in the elderly”. A worsening in RLS severity over three years was significantly associated with more depressive symptoms, worse sleep quality and lower quality of life. The short-term variation of the two key symptoms of RLS “urge to move” and “dysaesthesia” showed distinctive circadian patterns with rhythms that were strongly related to RLS severity.

An increase in RLS prevalence with age has been found in many cross sectional studies of the general population [1], [2]. No data have been published evaluating the long-term course of RLS symptoms beyond the duration of clinical trials. Satija and Ondo [14] described a progressive symptomatology with increasing age during the first seven to eight decades, with an improvement of RLS symptoms in the ninth decade. In contrast we found an association of severity with age only up to the age group 45–54 years. Beyond that group average symptom severity did not increase with higher ages in treated RLS participants.

60% percent of the study population did not experience a relevant symptom change over the course of three years. Almost every participant was on RLS medication at baseline and during follow-up including a single medication or combinations of up to five different drugs. However, 45.9% of the study participants reported a change of at least one RLS specific drug, i.e. either a stop or a new intake of a dopaminergic drug, dopamine receptor agonist, an opioid, an antiepileptic drug or a benzodiazepine.

Many studies have described an influence of RLS on quality of life [15]. Direct (dyseasthesia, urge to move) and indirect (sleep deprivation and disruption of sleep) effects of RLS negatively influence the self perceived health status. Our findings contrast results from clinical trials that indicate an improvement of quality of life scores with decreasing RLS severity [16]. This difference might be contributed to an effect of ageing in this cohort due to the already high mean age of 65.9 years in the cohort at baseline.

Depressed mood, social isolation as well as a decrease in self-reported mental health [1], [17] have been found in RLS cases. Cross-sectional studies have revealed an association of RLS severity with subjective sleep quality, but not with self-rated depressive symptoms [18]. In two studies, also applying the CES-D as a self-administered rating scale [3], [19] higher CES-D scores in RLS cases compared to non-affected individuals and higher CES-D scores in cases with more severe RLS were found. In the COR-Study high mean CES-D scores with no change over time indicate that the majority of study participants had constant clinically relevant depressive symptoms. Improvement or worsening of RLS severity went along with rather modest changes in depression scores of 2 to 4 points during 3 years of follow-up. Interestingly a worsening in RLS severity was associated with a change in the depression score that was twice as high as the one that was associated with an improvement of RLS severity.

The COR-Study participants estimated their sleep quality as moderately impaired. It is known that insomnia is a risk factor for depressive episodes and increases the likelihood of recurrence of depression [20], but depression may also cause insomnia. Sleep-related symptoms are relevant contributors to the diagnosis of depressive disorders and might thus be a connecting link, even the most important contributing factor between RLS and depression.

The COR-Study has several strengths and limitations. It includes a large number of study participants from two different countries. It prospectively assesses important disease characteristics and potential consequences in regular intervals over a long time establishing a true time sequence between severity and subsequent outcomes. Implementing a 7-day diary into one of the questionnaires enabled the documentation of short-term variation in symptom characteristics.

A limitation of this study is that all diagnoses, comorbidities and medications are self-reported since the study is conducted by mailed questionnaire. This might cause a misclassification of comorbidities in some cases due to insufficient knowledge about diagnoses among the participants. The COR-study is conducted among participants of RLS patient organizations, who most likely represent a selection towards RLS patients with a special health awareness compared to all RLS affected individuals. However, it cannot be ruled out that patients suffering from a higher degree of severity of RLS were less likely to participate in the study. In addition a misclassification of RLS status cannot be excluded. We addressed this problem by including a so-called “gold standard” group with RLS, comprising about 6% of the study population. The reported results for this “gold standard” group were not different from the other study participants, supporting the robustness of our findings.

In summary in large cohort of RLS patient organization members mean RLS severity was rather stable over the course of three years, indicating “little change”, despite considerable changes in individual RLS medications. A worsening in RLS severity caused small to moderate negative effects on quality of life, depressive symptoms and sleep quality. No evidence was found to support the hypothesis that age is a determinant of progression of RLS beyond age 55 years. More research into treatment wish and medication adherence among RLS patients is needed, to enable informed choices for affected individuals before starting long-term treatments.
